# A couple-based HIV prevention intervention for Latino men who have sex with men: study protocol for a randomized controlled trial

**DOI:** 10.1186/s13063-018-2582-y

**Published:** 2018-04-05

**Authors:** Omar Martinez, M. Isabel Fernandez, Elwin Wu, Alex Carballo-Diéguez, Guillermo Prado, Adam Davey, Ethan Levine, Brian Mattera, Nikki Lopez, Omar Valentin, Ashley Murray, Madeline Sutton

**Affiliations:** 1 Temple University’s School of Social Work, 1301 Cecil B. Moore Avenue, Ritter Annex, 505, Philadelphia, PA 19122 USA; 2 Nova Southeastern University, 2000 South Dixie Highway, Miami, FL 33133 USA; 3 Columbia University School of Social Work, 1255 Amsterdam Avenue, New York, NY 10027-5927 USA; 4 New York State Psychiatric Institute and Columbia University, 1051 Riverside Drive, Unit 15, New York, NY 10032 USA; 5 University of Miami, 1320 S Dixie Hwy, Coral Gables, Miami, FL 33146 USA; 6 University of Delaware, 540 S College Ave, Newark, DE 19713 USA; 7 GALAEI, 149 W Susquehanna Ave, Philadelphia, PA 19122 USA; 8 Centers for Disease Control and Prevention, Division of HIV/AIDS Prevention, National Center for HIV/AIDS Prevention, TB, Hepatitis, Epidemiology Branch, 1600 Clifton Road, MS E-45, Atlanta, GA 3029 USA; 9 Minority HIV/AIDS Research Initiative, Centers for Disease Control and Prevention, Division of HIV/AIDS Prevention, National Center for HIV/AIDS Prevention, TB, Hepatitis, Epidemiology Branch, 1600 Clifton Road, MS E-45, Atlanta, GA 3029 USA

**Keywords:** Couple-based HIV prevention intervention, HIV and AIDS, Health disparities, Implementation science

## Abstract

**Background:**

Latino men who have sex with men (MSM) experienced a 13% increase in HIV diagnoses from 2010 to 2014, more than any other racial/ethnic subgroup of MSM in the United States. If current HIV diagnoses rates persist, about one in four Latino MSM in the United States will be diagnosed with HIV during their lifetime. Although some efficacious HIV prevention interventions for Latino MSM exist, none have focused on couples. This paper describes the protocol of a randomized controlled trial (RCT) to test the preliminary efficacy of a couple-based HIV prevention intervention that is culturally tailored for Latino men and their same-sex partners.

**Methods:**

The RCT will determine the preliminary efficacy of Connecting Latinos en Pareja (CLP) to increase the proportion of anal sex acts that are HIV protected (i.e., anal sex acts in which condoms, pre-exposure prophylaxis (PrEP), treatment as prevention (TasP), or a combination thereof, are used to reduce risk of HIV transmission). CLP builds upon previous couple-based interventions with white and black MSM by incorporating biomedical prevention techniques, such as PrEP and TasP, implementing a framework responsive to the couple’s serostatus, and addressing the socio-cultural factors that influence HIV risk among Latino MSM. We also include input from community stakeholders, members of the target population, and a community advisory board as part of intervention development. Assessments will be conducted at baseline, and 3- and 6-months post-intervention to examine the intervention effects on outcomes (HIV-protected sex acts), and factors potentially mediating or moderating intervention effects.

**Discussion:**

This paper describes an innovative RCT that incorporates multiple HIV prevention techniques for Latino MSM in couples, regardless of serostatus. The ongoing involvement of community stakeholders, members of the target population, and a community advisory board is emphasized, and plans for widespread dissemination and application of findings into practice are discussed.

**Trial registration:**

Trial registration: NCT03048838. Registered on 3 February 2017.

**Electronic supplementary material:**

The online version of this article (10.1186/s13063-018-2582-y) contains supplementary material, which is available to authorized users.

## Background

Latinos, particularly Latino men who have sex with men (MSM), have been disproportionately affected by HIV infection. In 2014, 84% of all newly HIV-infected Latinos in the US were MSM [1], and current HIV surveillance data predict that, if current infection rates continue, one in four Latino MSM will be diagnosed with HIV in his lifetime [2]. Research suggests that experiences of stigma, discrimination, marginalization, sexual objectification, negative cultural perceptions of homosexuality, and cultural values such as “*familismo*” and “*machismo*” elevate vulnerability for HIV infection among Latino MSM [3].

Although efficacious interventions for Latino MSM have been developed [4, 5], the present intervention, *Connecting Latinos en Pareja* (CLP), is the only intervention developed specifically for Latino male couples, though it has yet to be rigorously and empirically evaluated. Findings from our formative study with Latino MSM suggest that the factors driving HIV risk for white male couples also operate among Latino male couples [6]. For instance, Latino men in male couples were more likely to report condomless anal sex and problematic alcohol use than those who were not in a relationship [6]. Developing and testing tailored interventions for Latino male couples is warranted to curb HIV infection among Latino MSM.

The present intervention, CLP, is an adaptation of a couple-based intervention for black male couples, *Connect ‘n Unite* [7]. CLP is novel in that it (1) integrates both biomedical prevention techniques (i.e., pre-exposure prophylaxis (PrEP) and treatment as prevention (TasP)) and psycho-educational skill building; (2) is adaptable for HIV prevention among both seroconcordant and serodiscordant couples; (3) employs an innovative algorithm for measuring HIV protection that goes beyond condom use as the sole indicator of HIV protection; and (4) is culturally tailored to Latino couples to address social, environmental, and contextual factors that intensify HIV risk in this population.

## Methods

### Theoretical underpinnings of CLP

Similar to CLP’s parent interventions, *Connect* and *Connect ‘n Unite*, CLP is grounded in social cognitive theory (SCT) and a relationship-oriented ecological framework [8, 9]. In concert with SCT, the content and activities utilized in CLP are designed to (1) provide information and knowledge to promote accurate risk appraisal; (2) build social and self-regulatory skills through problem solving, role plays, and other cognitive behavioral strategies; (3) increase self-efficacy to engage in HIV-protected sex and reduce risk behaviors; (4) increase positive outcome expectancies regarding HIV protection strategies; and (5) build and sustain social support networks for practicing HIV-protected sex. See Table 1 for constructs and measurements.

**Table 1:** Constructs and measurement instruments for Connecting Latinos en Pareja (CLP), 2017

Construct	Assessment Instruments
Primary Outcome (last 3 months)	Risk Behavior Assessment (adapted to include PrEP and TasP) [24]
Proportion of HIV-protected anal sex acts (condom, PrEP, TasP) with main male partner in last 3 months	AACTG Adherence Measure [25]
Secondary Outcomes	
Use of HIV protection (condom, PrEP, TasP) with most recent casual partner	Self-reported viral load and missed dose count
Number of casual sex partners in the last 3 months, including female partners	Visual Analog Scale for medication adherence [26]
Sero-sorting and strategic positioning	Sero-sorting and Strategic Positioning Questionnaire [27]
Smoking, alcohol, and substance use	Substance Use Inventory [24, 27]
Social Cognitive Factors	
HIV protection methods outcome expectancy	Condom Use Expectancies Scale [28]
HIV protection methods self-efficacy	Condom Use Self-Efficacy Scale [29]
Social support	Multidimensional Scale of Perceived Social Support [30]
Relationship-Oriented Ecological Factors	
Relationship attributes (e.g., length of time in relationship, engagement in extra-dyadic relationships)	Relationship Attributes [27]
Intimate partner violence	Intimate Partner Violence Screening Tool for Gay and Bisexual Men [31]
Relationship satisfaction and support	Same-Gender Couples Scale [32]
Communication	Communication Patterns Questionnaire [33]
Sexual communication	Sexual Communication Questionnaire [34]
Sexual relationship power	Sexual Relationship Power Scale [35]
Sexual satisfaction	Sexual Satisfaction Scale [36]
HIV-related dyadic measures	HIV-Related Dyadic Measures [27]
Social, Environmental, and Other Factors	
Sociodemographic variables (i.e., age, gender, education, income)	Sociodemographic Questionnaire [37]
Childhood sexual abuse	Childhood Sexual Abuse Assessment [37]
Psychological symptoms	Brief Symptom Inventory (BSI) [38]
Religiosity	Santa Clara’s Strength of Religious Faith Scale [39]
Acculturation	SASH Brief Scale [26]
Experiences of discrimination	Everyday Discrimination Scale [25]
Heteronormative indicators	Conservative/Traditional Moral Views in Sexuality Questionnaire [24]Gender Ideology Scale [40]Internalized Homophobia Scale [41]
Service system involvement	Services Review [24]
Neighborhood environment & safety	Social/Physical Order/Disorder Questionnaire [42]

The relationship-oriented ecological perspective refines SCT constructs and incorporates contextual, multilevel dynamics that influence risk among intimate partners. The ontogenetic level focuses on each partner’s developmental history and personal attributes (e.g., histories of childhood sexual abuse) and SCT constructs (e.g., PrEP use outcome expectancies) [8, 9]. CLP prompts participants to examine how their past experiences influence their current sexual practices. The microsystem consists of structural factors that are part of the immediate intimate relationship context in which sexual activity and risk/protective behaviors occur [8, 9]. CLP’s activities target communication and negotiation skills related to sexual risk behaviors, protection, and pleasure, as well as sexual decision-making, power, and control. CLP also addresses how the use of alcohol and other substances impairs cognition, which may undermine negotiation and mutual decision-making. See SPIRIT 2013 checklist (Additional file 1) and Fig. 2 for all study protocols.

The “exolevel” refers to external factors impinging on the immediate setting by acting as stressors or buffers on the likelihood of engaging in particular behaviors [8, 9]. CLP aims to strengthen social support and peer norms for having HIV-protected sex and limiting the use of alcohol and/or other drugs in sexual contexts. The “macrosystem” encompasses broad cultural values and belief systems (e.g., the “man” is the penetrator; if you disclose your sexuality or HIV status, your family will reject you) and stereotypes of Latino men (e.g., hot, desirable lovers; sex objects in the sexual marketplace) [8, 9]. CLP prompts participants to examine how these cultural values, beliefs, and stereotypes influence the couple’s sexual practices [9]. It also helps to build internal resources and external social support networks to reverse the objectification, rejection, and/or disenfranchisement that have been linked to HIV-risk behaviors [10].

### Study aims

The present study is guided by two aims:

Aim 1:To finalize and pilot test the CLP and control interventions and assessment measures, and to conduct preparatory activities to launch the randomized control trial (RCT).

Aim 2:To conduct a RCT (*n* = 150 Latino male couples) to test whether participants assigned to CLP report a higher proportion of HIV-protected anal sex acts with their main partners than those assigned to an attention control intervention, and to preliminarily examine the potential mediators and moderators of the intervention effects.

### Overview of study design

**Fig. 1:**
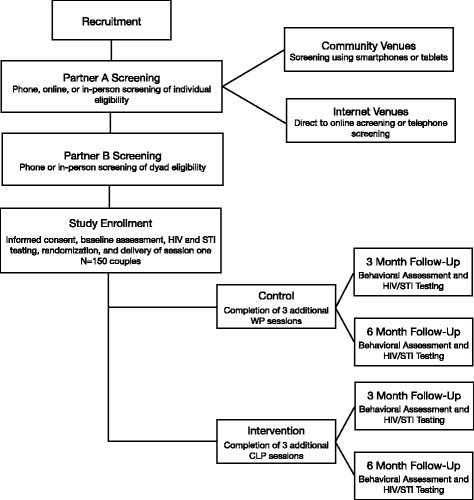
Study design schema for Connecting Latinos en Pareja (CLP), 2017

We will test the preliminary efficacy of CLP using a RCT design with two arms – the experimental intervention, CLP, and an attention control condition, Wellness Promotion intervention, which uses an identical couple modality and is comparable in time and attention [7]. The study will follow a two-phase timeline that coincides with the study aims. During phase one, intervention manuals and assessment measures will be developed, pilot tested, refined, and finalized. During phase two, we will recruit a sample of Latino male couples (*n* = 150 couples) and conduct a RCT to test the efficacy of the treatment intervention. Study phases and related activities are outlined in Fig. 1.

The study has been approved by the Institutional Review Boards of Temple University, the Philadelphia Department of Health, and the National Centers for HIV, Viral Hepatitis, STD, and TB Prevention’s project determination process. We have also obtained a certificate of confidentiality from the National Institute of Mental Health.

### Study sites

The primary assessment and project management activities will be conducted at Gay and Lesbian, Latino AIDS Education Initiative (GALAEI), a large Latino-serving community-based organization in North Philadelphia providing a range of services for LGBTQ Latinos, including HIV testing and prevention services, and services for people living with HIV. Recruitment and screening will be performed through community and internet venues. Intervention activities will be conducted at GALAEI, Temple University, and other safe spaces convenient to study participants.

### Community involvement and CAB

Active engagement of the host community has been identified as a ‘vital’ force in the implementation of successful, locally appropriate HIV prevention trials [11]. Throughout all phases of the study, input from the community and target population will inform the refinement and delivery of the intervention. During phase one of the study, we will convene a community advisory board (CAB) of 10 individuals, including key opinion leaders form the Latino MSM community, providers serving the health needs of Latino MSM, and community members. We will work closely with our community partners in identifying and selecting the CAB members to ensure that they are well-informed and able to provide the needed guidance.

Members of the CAB will be divided into two cohorts, each consisting of five members. Each cohort will participate in a CAB meeting once per year throughout the entire study duration. During phase one, the CAB will assist us in finalizing the interventions and assessments, identifying appropriate couples to participate in the refinement of the interventions, determining recruitment venues for the RCT, and other study-related activities. Two trained facilitators will present the goals, themes, key conceptual components, sample scenarios, role-plays, and key activities from the intervention sessions and solicit input from CAB members during audio-recorded sessions. The investigative team will audio-record the meetings and supplement them with transcribed meeting notes, then tag and group the information from each note by intervention content and activities. We will use this input to revise the interventions, while ensuring that we retain fidelity to the core components of the parent interventions.

Throughout phase two of the study (implementation of RCT), the CAB will convene and advise the investigative team on all aspects of the study, and will play a key role in reviewing ongoing study progress, developing plans to overcome challenges and handle adverse events, and interpreting and disseminating study findings. To date, CAB members’ feedback has been integrated into the refinement of study materials and has contributed to the formulation of a study logo and recruitment flyers.

### Couple pilot testing

To ensure that the interventions are culturally appropriate and relevant to the target population, phase one also involves a pilot test of the interventions and assessment materials with six male couples (in which at least one member of the dyad identifies as Latino/Hispanic). With the assistance of our CAB, GALAEI staff, and other community partners, we will identify 10 male couples that reflect the diversity of our future participants (e.g., concordant/discordant serostatus, racial/ethnic composition, age) and who are able to significantly contribute to the refinement of the intervention based on the initial interview. Of the 10 couples identified, six will be selected to pilot test the intervention, and four will act as alternates in case of attrition.

Once couples are recruited for pilot testing, a trained facilitator will administer the CAB-refined intervention sessions to three couples and couples will provide post-session feedback. Intervention sessions and review discussions will be audio-recorded and supplemented with transcribed notes; the facilitator will summarize the key themes. In addition to examining the intervention components (including activities and homework assignments) and reviewing language considerations, couples will provide input on logistics such as the structure of the intervention (e.g., length of time, frequency). The facilitator will group and summarize the material in a way that allows evaluation of the following:

- How comfortable were participants with the intervention content?
- What were the range and types of reactions to the specific content (e.g., communication and negotiation skills, HIV self-testing, PrEP, TasP, intimate partner violence, problematic alcohol use)?
- How did couple and relationship dynamics manifest throughout and affect the session?
- What are potential individual and dyad-level adverse reactions, if any?
- How well did the intervention relate to prominent issues among Latino male couples?
- How can the delivery style be enhanced with respect to cultural congruency and language use?

The team will incorporate refinements into the intervention based on the information provided and revise the session manuals accordingly. This process will be repeated with the remaining three couples, whose input will be used to make the final revisions to the sessions. The revised intervention will be presented to the members of our CAB, highlighting the revisions made. Once finalized, we will translate the manuals from Spanish to English. Couples who participate in this phase of the study will not be considered study participants, since they will serve in an advisory role, and any data collected from them will be used solely to refine the intervention, not to evaluate the intervention’s efficacy.

### Recruitment

We will recruit a sample of 150 male couples from community venues frequented by our study population (e.g., beaches, parks, gyms, coffee houses, clubs, community-based organizations, social clubs), internet venues and a study website, and online channels geared toward MSM (e.g. Grindr, Scruff, Jack’d). Across all venues, we will use methods such as (1) directly approaching men in community and internet venues, (2) making presentations at our community partners and special events, (3) obtaining referrals from community partners, (4) posting advertisements in selected internet and community venues, and (5) obtaining self-referrals or other referrals to recruit participants.

In both community and internet venues, trained bilingual staff will approach potential participants, describe the study, and obtain permission to screen for eligibility. In community venues, screening will be done electronically using smartphones or tablets; those engaged through internet venues will be asked to complete the formal screening via telephone or an online questionnaire.

Given that the study is couple-based, screening and eligibility determination will occur in two steps. First, the initial partner (Partner A) will complete the screening. He will be deemed ‘preliminarily eligible’ if he (1) meets demographic eligibility criteria (over 18 years old, identifies as a man who has sex with men, lives within the study area, identifies as Latino or reports having a partner who identifies as such) and (2) reports either two instances of HIV-unprotected anal sex within the relationship or at least one instance of HIV-unprotected anal sex with a male partner outside the relationship in the last 90 days. Operationalization of ‘HIV-protected’ and ‘HIV-unprotected’ anal sex acts will vary based on the couple’s serostatus, and will be determined by reported use of condoms, PrEP, and viral suppression (for HIV-positive individuals), or a combination thereof. If Partner A is eligible, the automated screening program will ask him to invite Partner B to complete the screening through the study website, telephone, or in person. Final eligibility determination of the couple will occur when Partner B completes the screener and the program determines whether or not the couple is eligible based on their linked data.

### Randomization to conditions

A computerized randomization program will be used to randomly assign couples to the treatment conditions with equal probability at the first study visit while they are completing the baseline assessment. Including both the baseline assessment and delivery of the first intervention session at the initial study visit minimizes potential biases in treatment effect estimates, due to differential attrition between randomization and the first session. It also ensures that all enrolled couples receive at least one intervention session.

### Intervention

The treatment intervention, CLP, consists of four sessions lasting 60–90 min administered to couples by a trained bilingual facilitator. Session content, scenarios, and examples will be adapted to each couple’s unique circumstances and HIV serostatus. During session one, couples will examine how cultural values (e.g., machismo) and couples’ dynamics and context impact sexual risks and health behaviors. In addition to providing basic information about HIV and AIDS, sexually transmitted infections (STIs), and substance use, couples are introduced to problem solving, the concept of couple’s self-care, and new prevention methods such as PrEP, while stressing those most relevant to the couple’s HIV status. At the end of session one, couples are asked to complete an assignment designed to enhance personal and couple-oriented self-care.

During session two, couples will be introduced to effective communication and goal setting skills, emphasizing how these skills relate to safer sex decision-making in order to increase motivation to utilize different prevention tools. Using examples relevant to the couple, the facilitator will use problem solving techniques to help the couple evaluate different prevention approaches and determine the most appropriate tools to incorporate into the couple’s risk reduction plan. Couples will be strongly encouraged to practice using at least one prevention tool in the coming week as part of their goal-setting and homework assignment.

In session three, couples will explore strategies for strengthening their relationships by (1) identifying and defining unwritten rules, (2) exploring the couple’s power and decision-making process, (3) examining triggers and influences leading to risky sexual behavior, and (4) developing action plans to increase sexual safety. The session incorporates skill-building roleplay scenarios for negotiating HIV-protected sex and exploring different prevention alternatives. By the end of the third session, couples are prompted to develop a couple-oriented relationship strengthening plan that includes alternative ways to practice safer and fun sex.

In the final session, facilitators will guide couples in identifying social support networks and resources for individuals living with HIV, within and outside the Latino and LGBTQ communities that could help attain their established goals. They will also review and refine their plans for engaging in HIV-protected sex, learn strategies for dealing with barriers to goal progress, and review key skills developed during the sessions. The session concludes with a “graduation” ceremony, during which couples make a commitment to follow the plan they developed.

### Control intervention

The comparison intervention is an attention-control (same duration and couple modality), four-session Wellness Promotion intervention [7] adapted to a Latino cultural context. Wellness Promotion focuses on identifying useful services, becoming accurate appraisers of reputable healthcare information from the internet and other sources, and advocating for equitable treatment and services from professionals. It is informational in nature and less interactive than CLP. It also contains ethically required information regarding HIV and STI prevention, including information and referral for PrEP, HIV-testing recommendations, as well as engagement in care and the importance of treatment adherence for HIV-positive participants.

### Quality assurance (QA) and fidelity monitoring

A variety of methods will be employed for QA and to ensure facilitator fidelity to the interventions. All sessions will be recorded using digital audio recorders; after each facilitator delivers a total of eight sessions, a random sample of 20% of each facilitator’s sessions for both conditions will be reviewed by a QA monitor. Upon review of session audio tapes, QA monitors will complete a QA Treatment Rating Form to assess facilitator fidelity with attention to time spent on each activity, adequacy of content delivery, occurrences and nature of treatment contamination, and clinical dynamics (e.g., relative balance of participation between partners). QA data are reviewed, processed, and addressed/troubleshot during regular facilitator supervision.

If QA data for a facilitator is found to fall in the insufficient range after attempting to rectify the problem, the facilitator will not be assigned new couples (but will continue with existing couples to ensure continuity for participants with all recordings reviewed) until the facilitator has successfully re-completed facilitator training. Any detected instance(s) of contamination by facilitators (i.e., overlap between conditions) or improperly handled responses to participants bringing up information from the other intervention will trigger immediate corrective action.

At the end of each session, facilitators will be required to complete a facilitator checklist to record attendance, the extent to which content was covered, time spent on each activity, whether unplanned content was introduced, and whether such content overlaps with the other condition. Additionally, participants will complete a Participant Feedback and Satisfaction form to elicit the extent to which participants experienced the facilitator as competent and the session as helpful, and to assess participants’ overall satisfaction with each session. Participants will also report the nature and frequency of contact with other study participants and whether they have been exposed to information from the other study arm; any evidence of contamination resulting from feedback forms, facilitator checklists, or issues raised in facilitator supervision meetings will be addressed immediately.

### Measures

Assessments will occur at baseline and 3 and 6 months post-intervention; assessments will be interviewer-administered and conducted at the individual level to ensure confidentiality.

#### Primary outcome: HIV-protected acts

The primary outcome, proportion of HIV-protected anal sex acts, will be assessed using a comprehensive risk behavior assessment that is adapted to the couple’s serostatus and use of prevention tools. Our team has developed an algorithm that incorporates three types of protective behaviors, namely (1) condom use, (2) PrEP utilization and adherence for those who are HIV negative, and (3) viral suppression (TasP) and antiretroviral treatment adherence for those who are HIV positive. In addition, the algorithm considers three additional elements, namely (1) couple’s HIV status (HIV negative seroconcordant, HIV positive seroconcordant, and serodiscordant), (2) whether either partner has had sex outside the relationship in the past 3 months, and (3) whether the couple reports 100% condom use with their main partner in the same period. Operationalization of HIV-protected sex acts is detailed in Table 2.

**Table 2:** Operationalization of HIV-protected sex acts based on couple’s serostatus, Connecting Latinos en Pareja (CLP), 2017

Algorithm for defining HIV-protected acts with main partner for participants reporting less than 100% condom use with main partner		
HIV status	No casual partners in last 3 months	One or both partners have casual partners
Seroconcordant negative	HIV protected	Both partners adherent to PrEP = protected
		Partner(s) having casual sex are/is adherent to PrEP = protected
Seroconcordant positive	Both adherent to antiretroviral therapy (ARV) and virally suppressed = protected	Both adherent to ARV and virally suppressed = protected
Serodiscordant	HIV-positive partner is virally suppressed and ARV adherent = protected	HIV-positive partner is having casual sex, is adherent to ARV and virally suppressed = protected
	HIV-negative partner adherent to PrEP = protected	HIV-negative partner is having casual sex and is adherent to PrEP = protected

Sexual behavior will be assessed separately for main male partners and casual male and female partners, if applicable. We will classify 100% condom use for anal insertive or anal receptive with main partner in the last 3 months as HIV-protected acts. For participants who report less than 100% condom use, we will assess whether those sex acts that were not protected by condoms were protected by PrEP (for HIV-negative seroconcordant or serodiscordant couples) or TasP (for HIV-positive seroconcordant or serodiscordant couples). Although the intervention is delivered at the dyad level, sexual risk will be assessed at the individual level, given the possibility that some couples who completed the intervention may not be together at either follow-up. Thus, assessments of sexual risk with primary partners will measure sexual behavior with participants’ current primary partner, whether or not this is the same partner who completed the intervention.

Among participants who report viral suppression or use of PrEP, medication adherence will be assessed by multiple measures to confirm the high probability of maintained viral suppression or PrEP protection, respectively, across those sex acts not protected by condom use. These measures include missed dose counts, a visual analog scale [12], and validated AIDS Clinical Trials Group adherence measures [13]. Although these assessments are based on self-report rather than biomarkers (i.e., viral load tests to confirm sustained viral suppression, blood tests to assess antiretroviral adherence), the proposed algorithm provides a comprehensive conceptualization of HIV protection that incorporates currently available prevention approaches.

#### Secondary outcomes

Secondary outcomes include HIV protection with male partners outside of the couple relationship (often referred to in the literature as ‘casual’ partners), chlamydia and gonorrhea infection, use of other HIV risk reduction strategies such as sero-sorting and strategic positioning, and smoking, alcohol, and other substance use. To assess HIV protection among casual male partners, we will ask participants the number of casual male partners in the last 3 months. To minimize recall bias, we will focus on the last anal sex encounter with a casual partner. Participants will report the type of anal sex and whether or not a condom was used from start to finish; we will also assess if it was protected via PrEP or TasP, and the self-reported HIV status of the casual partner. If the casual partner is HIV positive, we will ask if the partner is on treatment and if he is virally suppressed.

For those who consent to optional HIV and STI testing, we will test for current chlamydia and/or gonorrhea infection. Participants will be asked to collect a urine sample, throat swab, and rectal swab to detect genital, oral, and rectal infection, respectively. Those consenting to HIV and STI testing will also be tested for HIV by certified testers at the study site using a rapid HIV test.

We will also capture more informal HIV sexual risk reduction strategies, such as strategic positioning (choosing sex role in anal intercourse based on partners’ serostatus) and serosorting (selecting partners with concordant serostatus). Utilization of these risk reduction strategies will be assessed using a five-item, self-reported questionnaire that includes questions such as, “*In the past ninety days, did you intentionally have sex only with HIV-negative partners in order to reduce the risk of becoming infected with HIV?*” (if HIV negative) and, “*In the past ninety days, to reduce risk, did you only have insertive sex (i.e. you were the top) with HIV-positive men?*”

Smoking, alcohol, and substance use will be assessed using the 12-item Substance Use Inventory [14], a validated measure that assesses tobacco, alcohol, and illicit drug use in the participant’s lifetime and within the preceding 30 and 90 days.

#### Other measures

**Fig. 2:**
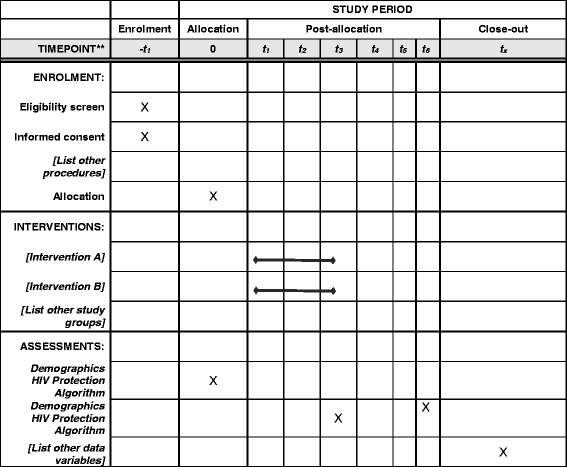
Schedule of enrolment, interventions, and assessments

Further measures will assess social cognitive factors, relationship-oriented ecological factors, and cultural and environmental factors that influence HIV risk or potentially moderate or mediate intervention effects. The majority of the measures are comprised of or based on validated scales that have been previously used in studies with Latino MSM, black MSM and/or previous research with couples. A complete list of study measures is outlined in Fig. 2.

### Data collection

All study data will be collected electronically using REDCap, a software application designed to build online surveys and databases. REDCap provides numerous safeguards against confidentiality breaches and is designed to comply with HIPAA, 21 CFR part 11 and FISMA regulations. Upon completion of assessments, data are automatically uploaded to a secure, password-protected cloud database; participant assessment data are not linked to identifying information. Data gathered from REDCap may be seamlessly imported into statistical software packages for subsequent data analysis.

### Data analysis

Initial data analysis tasks will involve univariate and bivariate statistics to characterize the sample and review the distributional properties of measures across treatment arms. To evaluate the efficacy of the intervention, we will use random effect models and/or generalized estimating equations (GEE) modeling 6-month follow-up differences, adjusting for characteristics at baseline, 3-months, and treatment arm in order to increase statistical precision. GEE models will consistently be used in cases where unobserved heterogeneity invalidates the application of random effect models. Additional covariates will be included as indicated by comparison of baseline differences and variables of theoretical importance (e.g., serostatus concordance). We will also explicitly test baseline × treatment interactions. We expect to model our main outcome as a continuous variable, but based upon examination of pilot data, some adjustment for interval censoring (i.e., considerable proportions reporting condom use on all or no occasions) may be required in order to meet distributional assumptions.

Following evaluation of Aim 2, we will perform a set of exploratory analyses to develop dyadic models for sero-discordant couples in which partners are not exchangeable in terms of risk [15]. We will also explore factors associated with the magnitude of treatment effects (i.e., potential moderators) as well as potential mediators. Effect modification will be evaluated directly through random effects models or as multiplicative composites in GEE models. Hypothesis testing will implement an intention-to-treat approach. We will employ GEE to account for the non-independence in measures arising from (1) “autocorrelation”, due to repeated measures with the same person, and (2) “intraclass correlation”, arising from partners in a dyad who are reporting on behaviors conjoint within that dyad.

#### Missing data

Missing data introduce uncertainty about unobserved values and their potential influence on parameter estimates and study conclusions [16, 17]. Application of recent developments in non-ignorable data (i.e., missing not at random) will include pattern mixture models, semiparametric models (e.g., inverse probability weighted), and stratification-based analyses [18, 19]. Sensitivity analyses will determine the extent to which conclusions depend on the assumptions about unobserved values [20]. Identification of missing data patterns will ensure that relevant covariates are included implicitly (i.e., auxiliary variables) or explicitly, as appropriate. Based on our previous research with Latino MSM, we do not expect attrition or missing data to be a significant issue; however, we are well prepared in the event that it occurs.

#### Power analysis

The sample size of 150 male couples (*n* = 300) was chosen to ensure sufficient power to detect differences in the proportion of HIV-protected sex acts between treatment conditions with a significance level of α = 0.05. Autocorrelation was estimated to be 0.15 and intraclass correlation was estimated to be 0.66; these estimates were derived from prior MSM couple-based intervention research studies for conjoint, condom-use variables [21]. We assumed a pre-intervention proportion of HIV-protected acts of 30%, based on pilot study data collected from Latino men in same-sex relationships. Power analyses also utilized an effect size involving a 12 percentage point increase in HIV-protected acts (i.e., a small-to-moderate effect size). Results indicate that 80% power is achieved for our primary outcome with a final sample size of 63 couples/arm; this extrapolates to a starting sample of 75 couples/arm (or a total of 150 couples) if we model the final sample size to include 15% attrition. Prior couple-based research found significant differences for behavioral outcomes with as few as 60 couples/arm [22, 23]. Actual power is likely to be higher due to (1) gains in efficiency via covariance adjustment, (2) analysis will use data provided by participants prior to their attrition (whereas power analyses assumed all data from an attrited participant would be unavailable), and (3) the likelihood of > 85% retention, based on the team’s successful retention in previous research with Latino male couples.

### Plans for dissemination

We hope that findings from this study will help reduce the impact of HIV on Latino male couples. We define dissemination as an ongoing, multi-directional process. Not only must these efforts be guided by science, but our approaches must be tailored to the intended audiences. Our anticipated audiences include (1) scientists, (2) prevention practitioners, (3) policy-makers, and (4) the community at-large. For the scientific community, we will use traditional dissemination vehicles, including manuscripts and presentations at international and national meetings. Given the study’s commitment to community and stakeholder involvement, we will work closely with our CAB and GALAEI to facilitate integration of findings into public health practice and the larger community.

Insights gathered from the first two CAB meetings have already served as the basis for an oral presentation at the 2017 American Public Health Association conference, focusing on the development of effective health communication strategies for recruiting and engaging Latino MSM. The aforementioned abstract details CAB members’ call for communication and recruitment materials that are culturally appropriate, are inclusive of in-group differences (including transgender individuals) and linguistic diversity, and avoid hypersexualized portrayals of Latino men, which several CAB members identified as a salient concern among current communication strategies targeting Latino MSM.

As the study progresses, we will elicit CAB members’ and agency staff and volunteers’ impressions of the study, ascertain their views on the pragmatic utility of the intervention, and generate ideas for further research and dissemination of findings. We plan to assist our local health department in planning initiatives and will explore other vehicles with our community partners to maximize utilization of our findings by local HIV prevention practitioners. Finally, we will present our findings to community-based organizations and their clients at seminars and workshops, and will work collaboratively to explore the relevance of the findings to their unique circumstances and settings.

## Discussion

The evidence indicating that HIV transmission often occurs within primary relationships [5, 6] suggests that focusing HIV risk reduction efforts on male couples may present a critical – albeit often overlooked – opportunity to reduce the spread of HIV among MSM. Moreover, the need for HIV prevention interventions to be culturally tailored and otherwise context sensitive is highlighted by a growing body of evidence supporting greater efficacy among such interventions [14]. The present study and related intervention make several advances in this area of HIV prevention, and build upon previous work in several innovative ways.

First, we focus on Latino MSM, an understudied and underserved group, disproportionately affected by HIV and AIDS and identified in the National HIV and AIDS Strategy as a group in need of targeted prevention efforts. Second, we are intervening at the level of the couple, where a high proportion of HIV transmission occurs. Intervening at this level offers a number of advantages over individual level interventions, and provides the opportunity to influence safer sex practices among two individuals simultaneously, to strengthen couple dynamics and relationship communication skills, in turn enhancing safer sex decision-making and negotiation skills, and to engage individuals who may not otherwise be targeted for individual-level prevention efforts, despite risk for HIV infection. These benefits have the potential to strengthen and enhance the broader public health impact of the intervention. Third, rather than developing an entirely ‘new’ intervention, we are refining an evidence-based HIV prevention intervention for black male couples to apply to Latino male couples. The resulting intervention, CLP, can thus advance more quickly through the testing process and be ready for widespread community implementation more rapidly. Fourth, we employ a novel outcome variable, conceptualizing ‘HIV protected acts’ to extend beyond condom use to include acts protected by PrEP and TasP.

### Limitations

Despite its unique advantages, there are several limitations. We will recruit a convenience sample, which limits generalizability. We acknowledge that the sample may not reflect the general population of Latino MSM. The budgetary constraints and the length of the project period of the MARI initiative limit our ability to include a 12-month follow-up. Thus, while we may detect change at 3 or 6 months post intervention, we will not be able to determine if the effects are sustained over time. Our sample size, although adequate to test for intervention effects, is not powered for efficacy. Thus, we cannot test the effects of moderators or mediators; moderation and mediation analysis are exploratory. Our primary outcome is limited to sexual activity with main partners. While appropriate for testing a couple-based intervention, we recognize that the HIV risk and protection with outside partners should also be considered. We will be conducting these as secondary analysis.

## Conclusion

Engagement of a CAB, rigorous measures testing, and systematic implementation processes are necessary prerequisites for the large-scale delivery of peer-led HIV interventions to improve HIV protection among Latino MSM. HIV prevention research trials that directly engage Latino MSM couples and key stakeholders are vital to inform culturally tailored interventions, improve HIV protection, and reduce HIV-related disparities that disproportionately affect Latino MSM. The present study not only advances HIV prevention research, but more importantly, if CLP results in significant behavior change, it holds promise in helping to reduce the HIV epidemic among Latino male couples.

## Trial status

We are currently recruiting study participants.

## Additional files

Additional file 1: SPIRIT 2013 Checklist: Recommended items to address in a clinical trial protocol and related documents. (DOC 120 kb)

Additional file 2: A couple-based HIV prevention intervention to promote HIV protection among Latino male couples. (DOCX 75 kb)

## Abbreviations

CAB: community advisory board
CLP: Connecting Latinos en Pareja
GEE: generalized estimating equations
MSM: men who have sex with men
PrEP: pre-exposure prophylaxis
QA: quality assurance
RCT: randomized controlled trial
SCT: social cognitive theory
STI: sexually transmitted infections
TasP: treatment as prevention
