# Supplementary material for: A couple-based HIV prevention intervention for Latino men who have sex with men: study protocol for a randomized controlled trial
Source: Trials. 2018 Apr 5;19:218. doi: 10.1186/s13063-018-2582-y (PMC5887179; doi:10.1186/s13063-018-2582-y)
Supplement: Supplementary file 2 — A couple-based HIV prevention intervention to promote HIV protection among Latino male couples. (DOCX 75 kb) [file 13063_2018_2582_MOESM2_ESM.docx]

**Additional file 1**


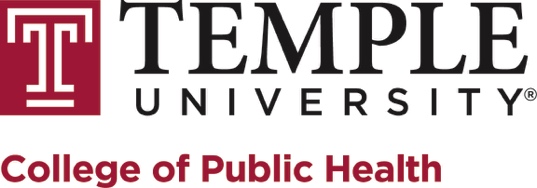


**Informed Consent**

***Title of Research*:** A Couple-based HIV Prevention Intervention to Promote HIV Protection among Latino Male Couples

***Version Date:*** December 12, 2017

***Investigator & Department:*** Omar Martinez, JD, MPH, MS

Assistant Professor, Temple University

College of Public Health, School of Social Work

***Funding Source:*** Centers for Disease Control and Prevention (CDC)

***Why am I being invited to take part in this research?***

You are being asked to participate in a research study. We are inviting you because you told us that you are in a relationship with a man and either you or your partner consider yourselves to be Latino or Hispanic. This form gives you important information about the study. It describes the purpose of this research study, and the risks and possible benefits of participating. If there is anything in this form you do not understand, please ask questions. Please take your time. You do not have to take part in this study if you do not want to. If you take part, you can leave the study at any time. In the sections that follow, the word “we” means research staff.

If you sign this form, you are agreeing to participate in this research study. You should not sign this form until you are sure you wish to participate, and all of your questions about this study have been answered.

***What should I know about this research?***

- Someone will explain this research to you.
- Whether or not you take part is up to you.
- You can choose not to take part.
- You can agree to take part and later change your mind.
- Your decision will not be held against you.
- You can ask all the questions you want before you decide.

***Who can I talk to about this research?***

If you have questions, concerns, or complaints, or think the research has hurt you, contact the research team at Omar Martinez, 1301 Cecil B. Moore Avenue, Ritter Annex, 505 Philadelphia, PA 19122, [omar.martinez@temple.edu](mailto:omar.martinez@temple.edu), 215-204-1223.

This research has been reviewed and approved by an Institutional Review Board. You may talk to them at (215) 707-3390 or e-mail them at [irb@temple.edu](mailto:irb@temple.edu) for any of the following:

- Your questions, concerns, or complaints are not being answered by the research team.
- You cannot reach the research team.
- You want to talk to someone besides the research team.
- You have questions about your rights as a research subject.
- You want to get information or provide input about this research.

You may also talk to the Research Participant Coordinator at the Philadelphia Department of Health IRB at (215) 685-0869 or [research.participant.DPH@phila.gov](mailto:research.participant.DPH@phila.gov) with any questions, concerns, or complaints.

***Why is this research being done?***

We are doing this research to test a new program, called Connecting Latinos en Pareja (CLP). This program will try to help male couples build stronger connections with each other and make healthier life choices. We are comparing this new program with the Wellness Promotion Program. That program focuses more on general health. We want to know if being in either the "Connecting Latinos en Pareja" or "Wellness Promotion Program" has any effect on helping men change the behaviors that put them at risk for HIV and other sexually transmitted infections (STIs).

***How many people will take part in this study?***

About 150 male couples in Philadelphia, Trenton, and nearby regions will take part in this study, so about 300 people total will take part in the study. At least one member of the couple must be Latino or Hispanic in order to take part in the study.

***How long will I be in this research?***

We expect that you will be in this research for about 7- 8 months.

***What happens if I agree to be in this research?***

Computer Survey:

If you agree to be in this study, we will ask you and your partner to set a date to come to our office. At that visit, each of you will complete a 60 to 90 minute survey on a computer. In the survey you will be asked to provide information about yourself and your relationship. You will also be asked about your sex practices and your use of alcohol and drugs, which might be sensitive. While you are completing the survey, you and your partner will be assigned by chance to be part of one of the two programs. This means you could either be assigned to “Connecting Latinos en Pareja” or “Wellness Promotion.”

Optional HIV/STI Testing:

If you agree to be in this study, we will ask you to HIV and STI tests. The HIV test is optional. However, you will have to agree to the STI test if you want to be part of the study. You also grant us access to receive your test results. If you and your partner opt to do these tests:

- Both of you will be tested for HIV from a drop of blood we will get from your finger.
- You will be given your HIV result that day in private.
- If you have a positive result on the HIV test, you will be referred for another HIV test at the Drexel Partnership to make sure this result is correct.

We will also test you for two other sexually transmitted infections. These are gonorrhea and chlamydia.

- You will be asked to provide a urine sample that will be used to test for these STIs.
- You will also be asked to give yourself a rectal swab. This will be used to test for these same STIs in your rectum.
- The urine sample and rectal swab you provide will be tested for gonorrhea and chlamydia by the Thomas Jefferson University. It will take a couple of days to process the tests at the lab.
- We will not be able to conduct oral swabs for gonorrhea and chlamydia.
- You will be informed of the results. We cannot provide you with medical care or treatment; however, we will provide you with information on free STD clinics, including Health Center 1. You might need to go through regular clinic procedures to be seen and treated.

Sessions:

After this first appointment, you and your partner will participate in 4 weekly sessions with a facilitator. Each session will last less than two hours. Most couples will complete each session in 60 to 90 minutes. If you are in the Connecting Latinos en Parejas program, the facilitator will provide information and ask you to complete activities, participate in games, and engage in discussions. You will talk about sensitive things including your relationship, sex, and how you talk with your partner. The goal of the program is to find ways to improve your relationship and your health. The Wellness Promotion program is very similar, except it focuses on general topics related to your health. The facilitator will present information on things such as nutrition, exercise, and stress, and you will participate in activities and engage in discussions.

The sessions will be audio-recorded so we can see how well the staff are providing the sessions. Only research staff will review the recordings. The recordings will be destroyed at the end of the study. You have the right to ask for any portion of the recording where you are talking to be erased. No one will question you about this.

At 3 and 6 months after you complete the last sessions, you will be asked to come back to our office to complete the survey. If you agreed to the HIV and STI testing, you will also be tested for HIV and gonorrhea and chlamydia again. If, at any point, any of these tests shows that you have an infection, we will refer you to appropriate care. The study will not pay for this care. However, we will provide you with information on free clinics that can provide treatment for STIs. Also, you will be provided with information about clinics providing care to HIV positive individuals at no cost.

***Is there any way being in this research could be bad for me?***

We will take every step possible to keep your information confidential, but we cannot guarantee total secrecy. Sensitive and personal information may come up in the sessions. This may lead some people to feel uncomfortable or anxious, and could cause unintended effects on your relationship. If you need help, the facilitator can give you information about where you can get help. You can contact your doctor or therapist if needed.

***Will being in this research help me in any way?***

You will not gain any direct benefits from participating in this study. The testing for HIV and other STIs may prove beneficial to you. You and your partner may learn things that may help to protect you from HIV, STIs, and other threats. We also hope that you will enhance and strengthen your relationship with your partner.

***Do I need to give my consent in order to participate?***

If you decide to participate in this study, you must sign this form. A copy will be given to you to keep as a record. Make sure you can commit to the time and responsibilities of being in the study when making your decision about participating in this study.

***What happens if I decide not to take part in this study?***

Participation in this study is voluntary. If you decide not to take part or if you change your mind later there will be no penalties or loss of any benefits to which you are otherwise entitled.

***What happens to the information collected for this research?***

To the extent allowed by law, we limit the viewing of your personal information to people who have to review it. We cannot promise complete secrecy. The Centers for Disease Control and Prevention (CDC), Temple University IRB, Philadelphia Department of Health IRB, Temple University, Temple University Health System, Inc. and its affiliates, and other representatives of these organizations may inspect and copy your information.

To protect your privacy, we will assign a unique number to you. We will not put your name (or any personal information) in study material. We will protect all study materials by keeping them in a locked file cabinet or in a password-protected computer. Only study staff will have access to this information.

To further help protect your privacy, we have obtained a Certificate of Confidentiality from the National Institute of Mental Health. The Certificate will help us to fight any demands for information that would identify you. This Certificate protects us from having to release your information, even if we get a court order or a subpoena. It does not prevent you, or any member of your family or social network, from voluntarily providing information about you or your involvement in this study. If you provide your consent, this Certificate will not prevent us from giving research information to an insurer or an employer. This means that you and your family must also actively protect your own privacy.

There are some exceptions to keeping information private. If you tell us that you may harm yourself or others or that elder abuse, child abuse, or sex with minors is occurring, we are required by law to notify the authorities.

The information collected from this study will be destroyed five years after the study is completed.

***Can I stop being in the study early?***

You can stop being in the study at any time. You do not have to give a reason.

Since this program is for couples, if you break up with your partner, neither you nor your partner can continue attending program sessions after your breakup. We cannot pay you for missed sessions. However, you will still be able to complete the remaining surveys separately.

***Who do I talk to if I want to stop being in the study?***

If you wish to stop being in this study at any point, contact the investigator:

Omar Martinez

1301 Cecil B. Moore Avenue, Ritter Annex Room 505

Philadelphia PA, 19122

(215) 204-1223

[omar.martinez@temple.edu](mailto:omar.martinez@temple.edu)

***Can I change my mind about the use of my personal information?***

Yes. You may change your mind and withdraw your permission to use and disclose your health information at any time. To take back your permission, you must tell the investigator, Omar Martinez, in writing. If you do not wish to mail the investigator, you may also take back your permission over email:

Mailing Address: 1301 Cecil B. Moore Avenue

Ritter Annex Room 505

Philadelphia PA, 19122

Email: [omar.martinez@temple.edu](mailto:omar.martinez@temple.edu)

In the letter/email, state that you changed your mind and do not want any more of your health information collected. The personal information that has been collected already will be used if necessary for the research. No new information will be collected. If you withdraw your permission to use your personal health information, you will be taken out of the study.

***What will I be paid for taking part in this research?***

If you agree to take part in this research, you will receive a token for your time and effort. After you complete the first survey and get tested for HIV, chlamydia, and gonorrhea (if you agree to the HIV and STI testing), you will receive $30. For completing the second survey and set of tests (if you agree to the testing), you will receive $40. For completing the last survey and set of tests (if you agree to the testing), you will receive $45. If you complete the second and last surveys and set of tests (if you agree to the testing), but your partner does not, you will still be paid. You will receive $25 for each of the four study sessions. You will only be paid if both you and your partner attend the session. We cannot pay you for missed sessions.

***Documentation of Consent***

The research study and consent form have been explained to you by:

_______________________________ __________________________________

Person Obtaining Consent Signature of Person Obtaining Consent

__________________________

Date

By signing this form, you are indicating that you have had your questions answered and you agree to take part in this research study. You are also agreeing to let Temple University use and share your health information as explained above. If you don’t agree to the collection, use and sharing of your health information, you cannot participate in this study. If you do not agree to the optional HIV, you will still be able to participate. However, you will have to consent to STI testing if you want to participate in the study.

_____ (initials) I agree to be part of this study.

_____ (initials) I agree to optional HIV testing, and agree to grant Temple
 University access to the results of the HIV/STI tests as explained above.

_____ (initials) I do not wish to take part in this optional part of the research.

______________________________________

Name of Subject (print)

______________________________________ ______________________

Signature of Subject Date
